# Nitrogen Limitation Alters Biomass Production but Enhances Steviol Glycoside Concentration in *Stevia rebaudiana* Bertoni

**DOI:** 10.1371/journal.pone.0133067

**Published:** 2015-07-20

**Authors:** Claire Barbet-Massin, Simon Giuliano, Lionel Alletto, Jean Daydé, Monique Berger

**Affiliations:** 1 Université de Toulouse, INP, EI Purpan, Département des Sciences Agronomiques et Agroalimentaires, 75 voie du TOEC, BP 57611, F-31076, Toulouse, France; 2 Université de Toulouse, INP, EI Purpan, UMR1248 AGIR, Département des Sciences Agronomiques et Agroalimentaires, 75 voie du TOEC, BP 57611, F-31076, Toulouse, France; Universidade Federal de Viçosa, BRAZIL

## Abstract

The need for medicinal and aromatic plants for industrial uses creates an opportunity for farmers to produce alternative crops. *Stevia rebaudiana* Bertoni, a perennial shrub originating from Paraguay, is of increasing interest as a source of zero-calorie natural sweeteners: the steviol glycosides (SVglys). The aim of this study was to investigate the relevance of nitrogen (N) supply for leaf yield and for SVgly concentrations in leaves, which are the two major components of *S*. *rebaudiana* productivity. In this regard, the relationship between leaf N concentration, CO_2_ assimilation, leaf production and SVgly accumulation was investigated. The experiments were conducted consecutively in growth-chamber (CC: controlled conditions), in greenhouse (SCC: semi-controlled conditions) and in field conditions (FC) on two genotypes. In CC and SCC, three levels of N fertilization were applied. Plants were grown on four locations in the FC experiment. Both N supply (CC and SCC) and location (FC) had a significant effect on N content in leaves. When light was not limiting (SCC and FC) N content in leaves was positively correlated with CO_2_ assimilation rate and biomass accumulation. Irrespective of the growth conditions, N content in leaves was negatively correlated with SVgly content. However, increased SVgly content was correlated with a decreased ratio of rebaudioside A over stevioside. The evidence that the increased SVgly accumulation compensates for the negative effect on biomass production suggests that adequate SVgly productivity per plant may be achieved with relatively low fertilization.

## Introduction

In the last decade, natural substitute sweeteners for sugar and synthetic sweeteners have received increasing interest owing to their low-calorie properties and to their potential dietary health benefits. Among the various sources of natural sweeteners, *Stevia rebaudiana* Bertoni, a perennial shrub of the *Asteraceae* family, native to limitrophe region between Paraguay and Brazil, is characterized by high concentration of steviol glycosides (SVglys) in its leaves, which are up to 200 to 400 times sweeter than sucrose. Commercial exploitation of *S*. *rebaudiana* started in the 1970s in Japan [1] and then extended to China, Asia, South America, and the USA [2– 4]. SVglys have been authorized as a food additive in the European Union since November 2011. Experimental cultivations in Europe have shown the ability of the specie to be cultivated in temperate climates [5, 6]. *S*. *rebaudiana* appears as a promising alternative culture in Europe, but additional investigations must be made to assess its cultivation plant requirements.

The SVgly content in *S*. *rebaudiana* leaves varies according to genotype, phenological stage, and growth conditions [2–4, 7]], at concentrations ranging from 10% to 30% of their dry mass [8]. It is generally accepted that SVgly content increases gradually up to the budding phase and the onset of flowering [9, 10]. Little research is currently available on how resource availability (mineral nutrition, light intensity) affects SVgly production in plants [11–14]. It was shown that the application of foliar nutrients [mainly KNO_3_ and Ca(NO_3_)_2_] led to an increase in chlorophyll, nitrogen, and potassium content in leaves but not in SVgly content [13]. In addition, an increase of light intensity promotes the leaf biomass production but has no significant effect on SVgly content [12]. However, for *S*. *rebaudiana*, the separate and combined effects of light intensity and mineral nutrition on plant physiology, growth, and SVgly production remain poorly understood. Yet progress in productivity is needed not only in terms of increasing the SVgly content, but also in terms of increasing the total leaf biomass and leaf to stem ratio.

**Fig 1:**
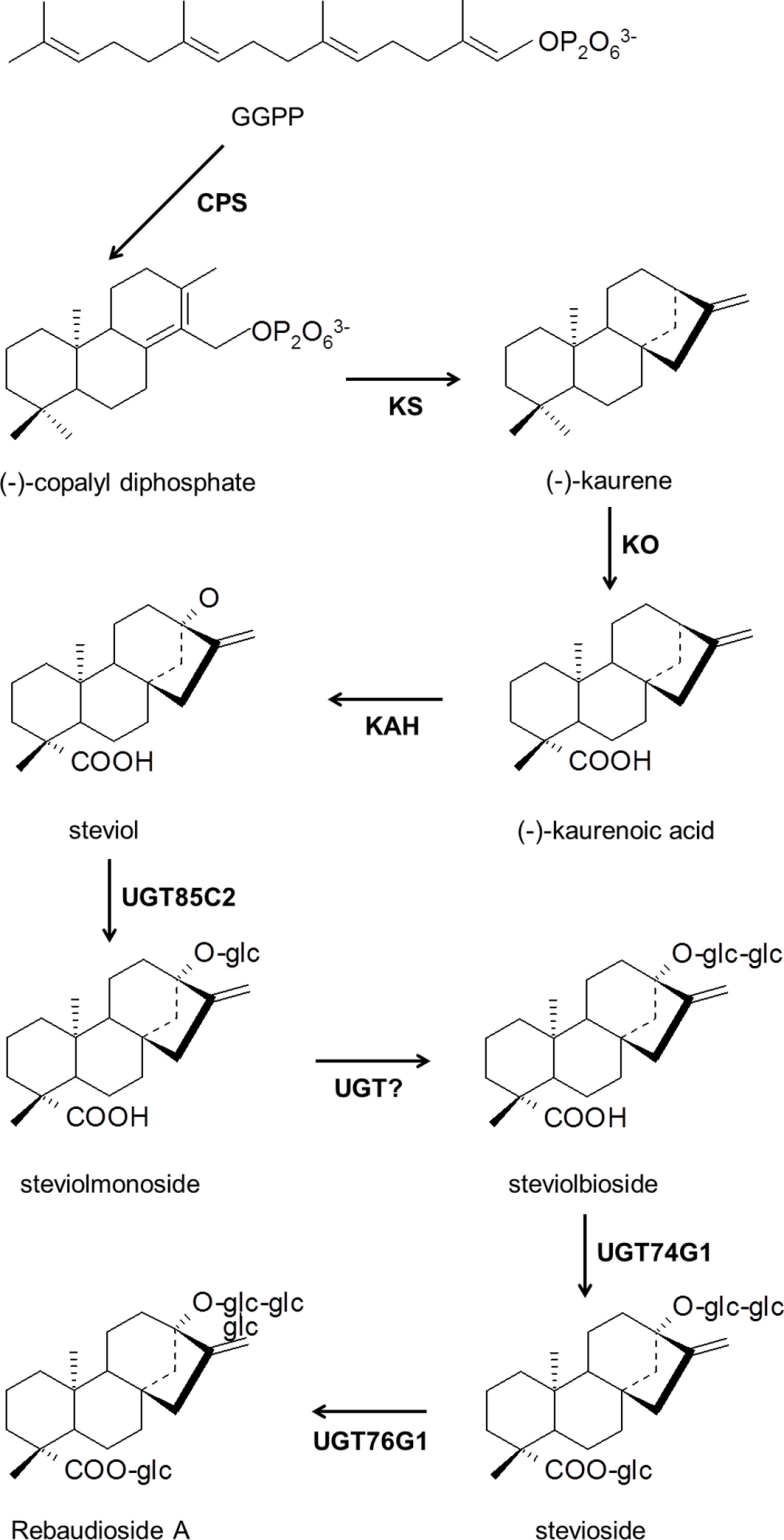
Biosynthetic pathway of steviol and its glycosides leading to rebaudioside A. GGPP, geranylgeranyl diphosphate; CPS, copalyl diphosphate synthase; KS, kaurene synthase; KO, kaurene oxidase; KAH, kaurenoic acid hydroxylase; UGTs, UDP-glycosyltransferases.

SVglys are glycosylated diterpenoids whose biosynthesis pathway is partly shared by gibberellins (GA) [15]. The accumulation of SVglys occurs in active photosynthetic tissues (mainly leaves). Steviol (SV), the diterpene aglycone moiety of SVglys, is synthetized via the 2-C-methyl-d-erythritol 4-phosphate (MEP) pathway, which occurs in the chloroplasts and whose precursors are pyruvate and glyceraldehyde 3-phosphate [16]. The resulting isopentyl diphosphate and dimethylallyl diphosphate are converted to geranylgeranyl diphosphate (GGDP). The first steps in producing kaurenoic acid from GGDP occur through the action of copalyl diphosphate synthase (CPS), kaurene synthase (KS), and kaurene oxidase (KO) [15] (Fig 1). Then the pathways leading to GA and SVglys diverge. Steviol is produced by hydroxylation of the kaurenoic acid at the C-13 position by the kaurenoic acid 13-hydroxylase (KAH) [17] (Fig 1). SV is then glucosylated, rhamnosylated, or xylosylated through the action of UDP-dependent glycosyltransferases (UGTs), to form the various SVglys [18] (Fig 1). The two major SVglys, stevioside (ST) and rebaudioside A (RA), account for more than 90% of the total glycoside content found in *S*. *rebaudiana* leaves [19]. ST is estimated to be 110–270 times sweeter than sucrose but has an astringent aftertaste. RA is reported as being 140–400 times sweeter than sugar and presents more pleasant sensory characteristics [5, 6].

The MEP pathway also supports the synthesis of photosynthetic pigments (chlorophylls and carotenoids) and hormones (gibberellins and abscisic acid) [20]. The two precursor molecules for the MEP pathway (G3P and pyruvate) are derived directly from photosynthesis or glycolysis. Not surprisingly, the MEP pathway is regulated not only by environmental signals but also by sugar levels [21]. Balancing the metabolism of C and N is crucial for plants [22]. Recently, it was shown in *Arabidopsis* that 4-hydroxy-3-methylbut-2-enyl diphosphate reductase (HDR), a key enzyme in the MEP pathway, depends not only on the photosynthetic carbon flux but also on NO_3_^−^-sensing systems [23]. Thus, a complex regulation of the MEP pathway must integrate both photosynthesis and NO_3_^−^ availability [23].

High concentrations of carbon-based secondary compounds (CBSCs) due to N deficiency have been observed in plants for a long time [24, 25]. To explain this phenomenon, the carbon-nutrient-balance (CNB) hypothesis [24, 26, 27] postulates a tradeoff between primary and secondary metabolisms. According to this hypothesis, plants preferentially allocate carbohydrates to growth so that more carbon is available for CBSC production only when, because of a lack of nutrients, plant growth is more restricted than photosynthesis [26]. Environmental conditions that would result in a high C/N ratio, and therefore to higher concentration of CBSCs, include high light levels, high inorganic carbon availability, and low nutrient availability [28]. This hypothesis is supported by results obtained on phenolic compounds [29–32] but is less consistent with observed accumulations of terpenoids in plants [32–34]. However, a high concentration of centellosides, which are terpenoids that accumulate in the leaves of *Centella asiatica*, was recently observed in plants subjected to N limitation [35].

In this study we hypothesized that changes in the balance between carbon and nitrogen can have opposite effects on biomass production and SVglys accumulation. Thus the relationships between CO_2_ assimilation, leaf N content, biomass production and SVgly content were studied in two experiments with different levels of N fertilization i) in a growth chamber where light limits maximum CO_2_ assimilation, ii) in a greenhouse with natural light. Two genotypes of *S*. *rebaudiana*, were chosen for their different SVgly profiles. Leaf N content, SVglys accumulation and biomass production were also measured on those genotypes in a four-location field experiment to test the effectiveness of these relationships in more realistic conditions of production.

## Materials and Methods

### Plant material

Two genotypes of *S*. *rebaudiana* were chosen from a population of “Criola” (a native population originating from Paraguay). These two genotypes have been studied since 2010 at the Agricultural and Food Sciences Department (INP-EI Purpan) at the University of Toulouse, France. Their SVgly composition is highly stable: genotype A contains ST and RA, genotype B contains ST but not RA. Both genotypes contain other less concentrated SVglys. The plants used in the experiments reported herein were obtained from stem cuttings taken from two-year-old parental plants.

### Experiments and growth conditions

#### Controlled and semicontrolled conditions

The same experimental procedures were applied to both experiments under controlled conditions (CC, growth chamber) and semicontrolled conditions (SCC, greenhouse). The experiments were conducted at the INP-EI Purpan, France (43°36'N, 1°24'E). Four weeks before the experiments, the plants (24 per genotype) were transplanted into three-liter pots containing loam (12:14:24 NPK kg m^-3^). At the beginning of the experiments, the plants were pruned, leaving 5 cm stems. From January to March 2013, the experiment was conducted under CC, in a growth chamber with 200 W of lighting supplied by high-pressure sodium (HPS) lamps, 200 W of lighting supplied by metal-halide (MH) lamps, and 400 μmol m^−2^ s^−1^ of photosynthetically active radiation (PAR) supplied at the plant level. The photoperiod was 16 h with 23°C/18°C day/night temperatures. The relative humidity (RH) was comprised between 70 and 80%. From May to July of 2013, the experiment was replicated under SCC, in greenhouse, with PAR of 1000 to 2000 μmol m^−2^ s^−1^. Day length was comprised between 14 and 15 h. The air temperature was comprised between 10 and 32°C during the day and 10 and 24°C at night. The RH fluctuated between 50 and 80%. At the end of both experiments (10^th^ week), the plants had reached the flower-budding stage.

Both genotypes (A and B) were treated with three levels of N (N1, N2, and N3, which corresponds to low, medium, and high levels of N, respectively; see Table 1 for composition and quantity). The plants were also supplied with P and K and a micro-element solution containing 0.01% B, 0.003% Mo, 0.001% Co, 0.01% Fe, 0.025% Mn, 0.01% Zn, and 0.01% Cu (B’Essentials, General Hydroponics Europe, France) enriched with Mg. Plants were daily irrigated (75–150 mL pot^-1^, depending on plant size and temperature) to avoid water stress. The fertilizers were given weekly. The experiments were arranged in randomized complete block design with two blocks and four replications by block.

**Table 1:** Composition of fertilizing solution and quantities supplied for experiments under controlled conditions (CC and SCC).

Mineral element	Concentration (g L^-1^)	Solution composition	*Volume supplied by week per plant (mL)*		
			N1	N2	N3
N	60	NO_3_^-^; NH_4_^+^, CO(NH_2_)_2_	0.18	1.575	3.15
Equivalent N (kg ha^-1^, density of 65,000 pl ha^-1^)			7	60	120

#### Field conditions

The experiment was carried out during one growing season (2014), in four experimental sites all located in south-west France: sites I and II (43°54'N, 1°41'E) in the region of Tarn, sites III (43°36'N, 1°24'E) and IV (43°29'N, 1°14'E) in the region of Haute-Garonne. The four locations were private lands, the experiments were authorized by the owners. No specific permissions were required for these activities. These field studies did not involve endangered or protected species. The characteristics of the four experimental fields are detailed in Table 2. In each site the experiment was laid out in a randomly block design with three blocks. Each plot consisted of 60 plants from the same genotype. Plant density was 6.5 pl m^-2^, with inter-row and intra-row spacing of 0.5 x 0.3 m on site I; 10 pl m^-2^ (0.5 x 0.2 m) on site II; 6.5 pl m^-2^ (0.5 x 0.3 m) on site III and 8 pl m^-2^ (0.4 x 0.3 m) on site IV. Plants were transplanted in the fields in May 27^th^-30^th^ 2014 and harvested at flower budding stage, in mid-September.

**Table 2:** Characteristic of the four location sites for experiments under field conditions.

Climatic characteristics from transplanting to harvest	Field I	Field II	Field III	Field IV
T mean (°C)	20.8	20.8	20.3	20.4
T max (°C)	27.5	27.5	26.1	25.5
T min (°C)	14.1	14.1	15.0	13.0
Cumulative degree-day (°C) ^a^	1705	1705	1674	1653
Rainfall (mm)	204	204	231	199
Field characteristics				
Slope and orientation	5% N	0%	0%	0%
Sand (2–0.05 mm %)	38.9	25.0	35.7	32.6
Silt (0.05–0.002 mm %)	25.8	60.0	47.4	49.2
Clay (< 0.002 mm %)	35.3	15.0	14.5	16.1
pH(H_2_O)	8.3	6.50	6.9	6.25
Organic matter (%)	1.69	2.50	2.60	2.05
Preceding crop	Fallow	Stevia	Fallow	Maize
Irrigation (mm)	20	10	160	120
Fertilization	None	None	None	None

^a^ $\text{Cumulative degree}-\text{day}=\overset{\text{n}}{\underset{\text{day}=1}{\sum }}\frac{\text{Tmax}+\text{Tmin}}{2}-\text{Vegetation zero}$; with vegetation zero fixed at 6°C

### Sampling and harvest analysis

The aerial biomass of each plant was harvested. Leaves and stems were separated by hand and oven dried at 40°C for at least 48 h. Three yield component indicators were measured: the total dry-plant biomass, the leaf biomass (g DM pl^-1^, where “DM” means “dry matter”), and the leaf-mass ratio (LMR, %), which is the fraction of the total-plant biomass (leaves + stems) due to leaves. For experiments under controlled (CC) and semicontrolled conditions (SCC), the leaf area (cm^2^), leaf biomass (g), and specific leaf weight (SLW, g m^-2^) were collected from the fully expanded leaves used for gas-exchange measurements.

### Nitrogen content in leaves

For each sample, 100 mg of dried and finely ground leaves were placed in a tin capsule. Their N concentration (% per dry mass) was determined by using a Thermo Scientific FLASH 4000 Nitrogen/Protein analyzer (Thermo Fisher Scientific Inc., USA). N concentration was used to compute the specific leaf N content (SLN, g m^-2^) for plants grown under CC and SCC.

### Steviol glycoside content and composition in leaves

100 mg of dried and finely ground leaves were extracted with 10 mL water at 50°C for 30 min. After centrifugation (7000 rpm, 5 min) and filtration (0.2 μm), 20 μL of the resulting solution were injected into a high-performance liquid-chromatography system on a C18 column (250 x 4.6 mm inner diameter, 5 μm particle size; Luna C18 Phenomenex, USA). SVglys were eluted by using an isocratic phase of ACN 31%: H_2_O (pH = 2.6, HCOOH) 69%, at 1 mL min^-1^ flow rate for 30 min. The SVglys (including ST, RA, and five minor SVglys) were detected at 200 nm. For quantification, stevioside was used as an external standard. Results were expressed as percentage per unit dry mass for the SVgly total content and as percentage of SVgly total content for the SVgly (ST, RA and minor SVglys) proportions.

### Leaf gas-exchange measurements

These measurements were made under CC and SCC only. The parameters measured were the maximum net rate of CO_2_ assimilation, *A*_max_ (i.e., the light-saturated CO_2_-assimilation rate at ambient CO_2_ concentration), the light-saturation point *I*_k_ (i.e., the light intensity at which *A*_max_ is reached), the dark respiration rate *R*_d_ (i.e., the respiratory carbon loss), and the apparent quantum yield of photosynthesis Φ_I0_ (i.e., the efficiency with which absorbed photons are converted into fixed carbon) [36]. In both experiments, light saturation curves for photosynthesis (*A* vs *Q*) were acquired at the flower-budding stage from fully expanded leaves by using a portable LI-6400XT system (Li-Cor, Lincoln, NE, USA). The ambient CO_2_ concentration *C*_A_ of the cuvette in the open gas-exchange chamber was precisely regulated at 400 μmol CO_2_ mol^-1^ by using a CO_2_ injection mixer. The leaves were maintained at the ambient temperature (23°C for CC, 20–25°C for SCC) in the open gas-exchange chamber, with a relative humidity between 50% and 70%. Measurements were made at decreasing photosynthetic photon flux densities (PPFDs) of 2000, 1500, 1000, 500, 250, 120, 60, 30, 15, and 0 μmol m^−2^ s^−1^. When needed, the measurements were started at an intermediate PPFD (500 or 1000 μmol m^−2^ s^−1^), which was then increased to 2000 μmol m^−2^ s^−1^ to acclimatize the leaves to high light intensities. The excel routines developed by Lobo et al. [37] were used to fit the resulting light response curves to a rectangular hyperbola from the following Michaelis—Menten-based model [38]:

$$P_{N}=\frac{\Phi_{I0}\times I\times A_{gmax}}{\Phi_{I0}\times I+A_{gmax}}-R_{d}$$ (1)

where *P*_N_ is the net photosynthesis rate in μmol(CO_2_) m^−2^ s^−1^, Φ_(I0)_ is the apparent quantum yield at *I* = 0 in μmol(CO_2_) μmol^-1^(photons), *I* is the photosynthetic photon flux density in μmol(photons) m^−2^ s^−1^, *A*_max_ is the maximum gross photosynthesis rate in μmol(CO_2_) m^−2^ s^−1^, and *R*_d_ is the dark respiration rate in μmol(CO_2_) m^−2^ s^−1^.

### Statistical analysis

The R software (R.2.15.1) [39] was used to perform the statistical analyses. A two-way complete analysis of variance was used for both experiments under CC and SCC, to determine if the N supply (N), genotype (G), and their interactions affected biomass and leaf production, SVgly content, and associated physiological parameters. A two-way complete analysis of variance was performed for the experiment under field conditions (FC), to determine if the location sites (LS), genotype (G), and their interactions affected biomass and SVgly accumulation. Significant differences between the treatments were determined by using Fisher’s least significant difference (LSD) test from the R package agricolae. The coefficient of determination *R* ^*2*^ was used to establish the relationship between plant biomass, SVgly accumulation and nitrogen content in leaves.

## Results

### Nitrogen content in leaves

**Fig 2:**
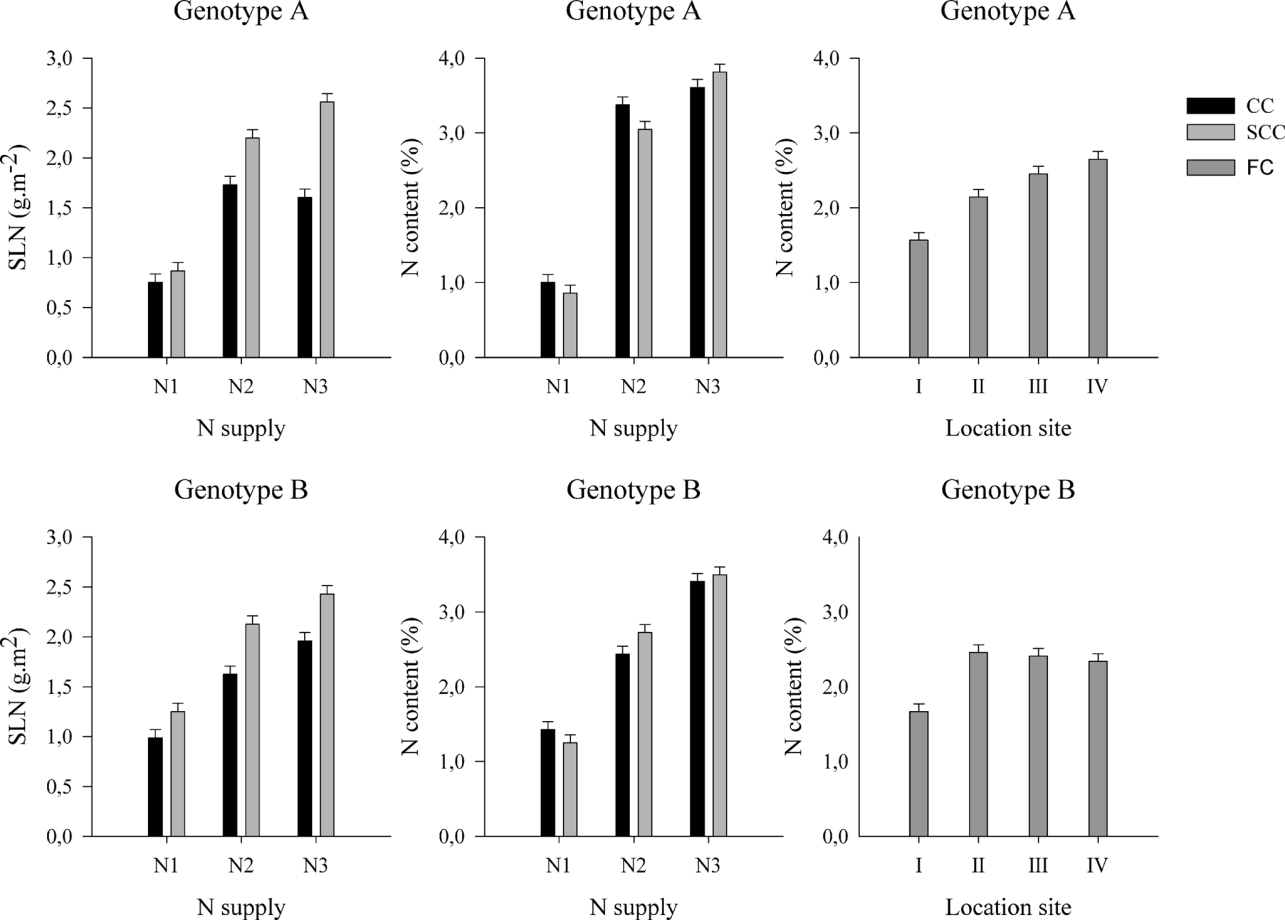
Specific leaf nitrogen (SLN, g m^-2^; mean±SE) and nitrogen content in leaves (N content, %; mean±SE) for genotypes A and B in controlled (CC), semi-controlled (SCC) and field conditions (FC) at different levels of fertilization (N1, N2, N3) or in different location sites (I, II, III, IV).

Under both CC and SCC, N content in leaves (%) and SLN (g m^-2^) were significantly lower under N1 supply than under N2 and N3 supplies (Fig 2). For each N supply, N content in leaves reached the same mean values in both experiments. However, for both genotypes, SLN was lower under CC compared to SCC under high N-supply levels (N2 and N3). Indeed, SLN takes into account the leaf size and thickness: the specific leaf weight (SLW, g m^-2^) was higher for plants grown under SCC at high N-supply levels (N2 and N3) compared to those grown under CC (data not shown). Under FC, the location site (LS) and the G x LS interaction influenced N content in leaves, which was significantly lower for the plants grown in field I than in the field II, III and IV (Fig 2).

### Yield components

#### Biomass production

**Fig 3:**
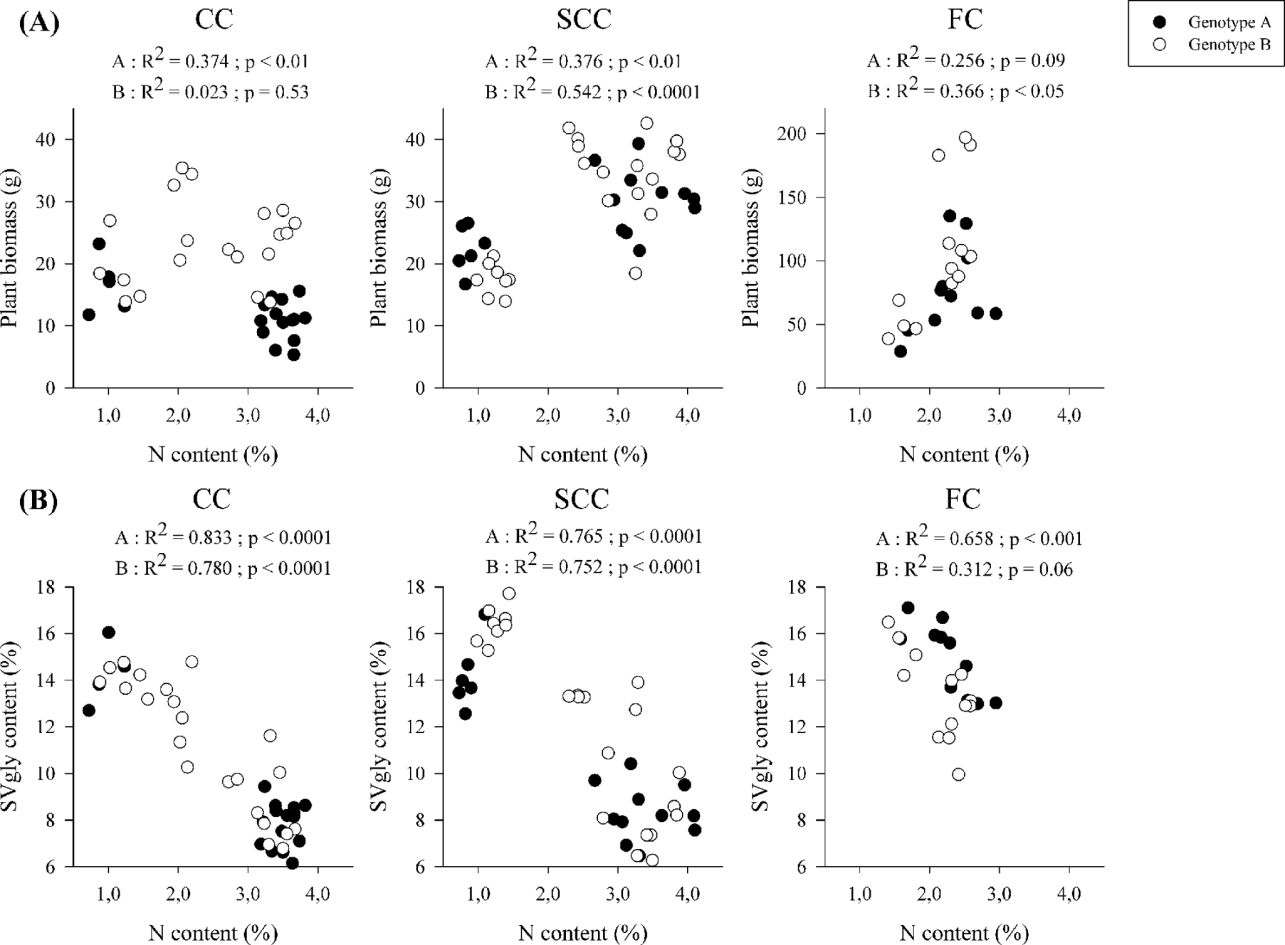
(A) Relationship between nitrogen content in leaves (N content, %) and plant biomass (g DM) and (B) Relationship between N content (%) and steviol glycoside content in leaves (SVgly content, %) for genotypes A and B in controlled (CC), semi-controlled (SCC) and field conditions (FC).

For both genotypes, no significant differences were obtained at the three different levels of N supply for plant and leaf biomass (g DM) under CC. In contrast, under SCC, leaf biomass increased with N supply for both genotypes (Table 3). Under FC, for both genotypes, the biomass production per plant was significantly higher on site III, and significantly lower on site I (Table 3). Even if other environmental conditions may have influenced biomass accumulation, the relationship between N content in leaves and plant biomass (Fig 3) shows it generally increased with N content in leaves (with exception of genotype A under CC).

**Table 3:** Yield components (mean ± SEM with SEM calculated from the ANOVA residual mean square with 28 df under CC, 32 df under SCC and 16 df under FC) as affected by genotype (G) = A and B and nitrogen supply (N) = N1, N2, and N3 or location site (LS) = I, II, III, IV.

Growth conditions (GC)	Genotype (G)	Nitrogen supply/Location site (LS)	Plant biomass (g DM)	Leaf biomass (g DM)	LMR (%)	SVgly content (% DM)	SVgly yield (g pl^-1^)	*N*
CC	A	N1	16.6 ± 2.3	11.6 ± 1.4	70.3 ± 2.0	12.8±0.6	1.6±0.2	5
		N2	12.3 ± 2.0	9.0 ± 1.2	74.2 ± 1.7	7.4±0.5	0.7±0.2	7
		N3	9.5 ± 2.0	7.0 ± 1.2	73.4 ± 1.7	8.0±0.5	0.6±0.2	7
	B	N1	21.0 ± 2.1	12.7 ± 1.3	60.3 ± 1.8	14.1±0.6	1.8±0.2	6
		N2	23.8 ± 1.8	14.5 ± 1.1	61.3 ± 1.6	11.2±0.5	1.6±0.2	8
		N3	24.1 ± 2.0	14.5 ± 1.2	60.4 ± 1.7	7.9±0.5	1.1±0.2	7
SCC	A	N1	21.3 ± 2.0	14.2 ± 1.2	66.6 ± 1.7	13.6±0.5	2.0±0.2	7
		N2	28.3 ± 1.8	18.1 ± 1.1	64.1 ± 1.6	8.1±0.5	1.5±0.2	8
		N3	32.3 ± 2.3	20.9 ± 1.4	64.8 ± 2.0	8.5±0.6	1.8±0.2	5
	B	N1	17.5 ± 1.8	11.3 ± 1.1	64.4 ± 1.6	16.4±0.5	1.9±0.2	8
		N2	32.9 ± 1.8	20.3 ± 1.1	62.5 ± 1.6	12.5±0.5	2.6±0.2	8
		N3	36.3 ± 1.8	21.0 ± 1.1	57.8 ± 1.6	7.8±0.5	1.7±0.2	8
FC	A	I	37.6 ± 7.1	21.3 ± 2.4	59.9 ± 1.7	16.5±0.5	3.5±0.5	3
		II	69.9 ± 7.1	31.6 ± 2.4	45.7 ± 1.7	16.2±0.5	5.1±0.5	3
		III	122.3 ± 7.1	55.7 ± 2.4	45.7 ± 1.7	14.5±0.5	8.1±0.5	3
		IV	63.2 ± 7.1	37.3 ± 2.4	59.4 ± 1.7	13.2±0.5	5.0±0.5	3
	B	I	54.8 ± 7.1	27.1 ± 2.4	49.9 ± 1.7	15.0±0.5	4.1±0.5	3
		II	97.9 ± 7.1	41.8 ± 2.4	43.0 ± 1.7	13.8±0.5	5.8±0.5	3
		III	190.4 ± 7.1	67.8 ± 2.4	35.7 ± 1.7	12.5±0.5	8.4±0.5	3
		IV	98.4 ± 7.1	42.5 ± 2.4	43.3 ± 1.7	11.2±0.5	4.8±0.5	3

*N* represents the number of plants under CC and SCC, and the number of plots under FC. CC, controlled conditions; SCC, semicontrolled conditions; FC, Field conditions.

The effect of genotype on the LMR (p < 0.01) was significant under the three growth conditions. LMR was higher for genotype A (73% under CC, 65% under SCC and 53% under FC) than for genotype B (60% under CC, 62% under SCC, 43% under FC). Under CC the LMR was not affected by N supply, while under SCC the mean LMR value of genotype B was significantly lower under N3 supply than under N1 supply (Table 3). Similarly, for both genotypes under FC, the lower LMR value was achieved on site III, where the plant biomass was the highest (Table 3).

#### Steviol glycosides content

For both genotypes, SVgly concentration in the leaf decreased with increasing N concentration. SVgly content decreased up to 42% for genotype A and 44% for genotype B with N2 and N3 compared to N1-N supply level under CC. It decreased by up to 40% for genotype A and 52% for genotype B Under SCC,. Under FC, plants grown on site IV (2.55% mean N content in leaves) were 20% (genotype A) and 26% (genotype B) lower in SVgly content than those grown on site I (1.62% mean N content in leaves) (Table 3). The relationship between N content and SVgly content in leaves was significant under the three growth conditions for genotype A and under CC and SCC for genotype B (Fig 3).

#### Steviol glycoside production per plant

The increase in leaf biomass upon increasing N-supply level almost entirely balanced the decrease in SVgly content under CC and SCC, resulting in a nonspecific response of SVgly production (g pl^-1^; Table 3). However, under FC, the increased plant and leaf biomass on site III resulted in a significantly higher SVgly production for both genotypes on this site (Table 3).

### Plant physiology and photosynthetic capacity

#### Light response curves

For both genotypes under CC, *A*_max_ and *R*_d_ had essentially the same values regardless of N-supply levels, while Φ_I0_ significantly decreased with enhanced nitrogen supply (Table 4). Under SCC, *A*_max_ for genotype A increased by 46% and 36% with N3 and N2 N-supply levels, respectively, compared with the N1 N-supply level. This effect was not observed for genotype B. For both genotypes, a high supply of N (N3) resulted in a significant increase in *R*_d_. No statistically significant differences occurred in Φ_I0_ due to the N different treatments (Table 4). For both genotypes, *I*_k_ was higher under SCC than under CC (Table 4). This increase was generally accompanied by an increase of *A*_max_ and *R*_d_.

**Table 4:** Parameters values for light-response curves (mean ± SEM with SEM calculated from the ANOVA residual mean square with 33 df under CC and 32 df under SCC) as affected by genotype (G) = A and B and nitrogen supply (N) = N1, N2, and N3.

Growth conditions (GC)	Genotype (G)	Nitrogen supply (N)	A_max_ μmol_(CO2)_ m^−2^ s^−1^	I_k_ μmol_(photons)_ m^−2^ s^−1^	Φ_I0_ μmol_(CO2)_ μmol_(photons)_^−1^	R_d_ μmol_(CO2)_ m^−2^ s^−1^	*N*
CC	A	N1	11.9 ± 2.3	1018 ± 102	0.078 ± 0.005	1.40 ± 0.18	8
		N2	11.4 ± 2.3	1146 ± 102	0.047 ± 0.005	1.58 ± 0.18	8
		N3	16.1 ± 2.5	1487 ± 109	0.062 ± 0.005	1.51 ± 0.19	7
	B	N1	14.2 ± 2.3	1112 ± 102	0.084 ± 0.005	1.25 ± 0.18	8
		N2	18.5 ± 2.5	1556 ± 109	0.069 ± 0.005	1.79 ± 0.19	7
		N3	18.4 ± 2.5	1562 ± 109	0.066 ± 0.005	1.69 ± 0.19	7
SCC	A	N1	24.7 ± 2.5	1791 ± 109	0.090 ± 0.005	2.04 ± 0.19	7
		N2	33.7 ± 2.3	1926 ± 102	0.089 ± 0.005	2.48 ± 0.18	8
		N3	36.1 ± 2.7	1950 ± 118	0.078 ± 0.006	2.98 ± 0.20	6
	B	N1	26.6 ± 2.3	1891 ± 102	0.079 ± 0.005	2.16 ± 0.18	8
		N2	29.7 ± 2.5	1900 ± 109	0.076 ± 0.005	2.44 ± 0.19	7
		N3	30.5 ± 2.3	1749 ± 102	0.080 ± 0.005	2.90 ± 0.18	8

*N* represents the number of plants under CC and SCC. CC, controlled conditions; SCC, semicontrolled conditions.

#### Relationship between specific leaf nitrogen and maximal rate of CO_2_ assimilation

**Fig 4:**
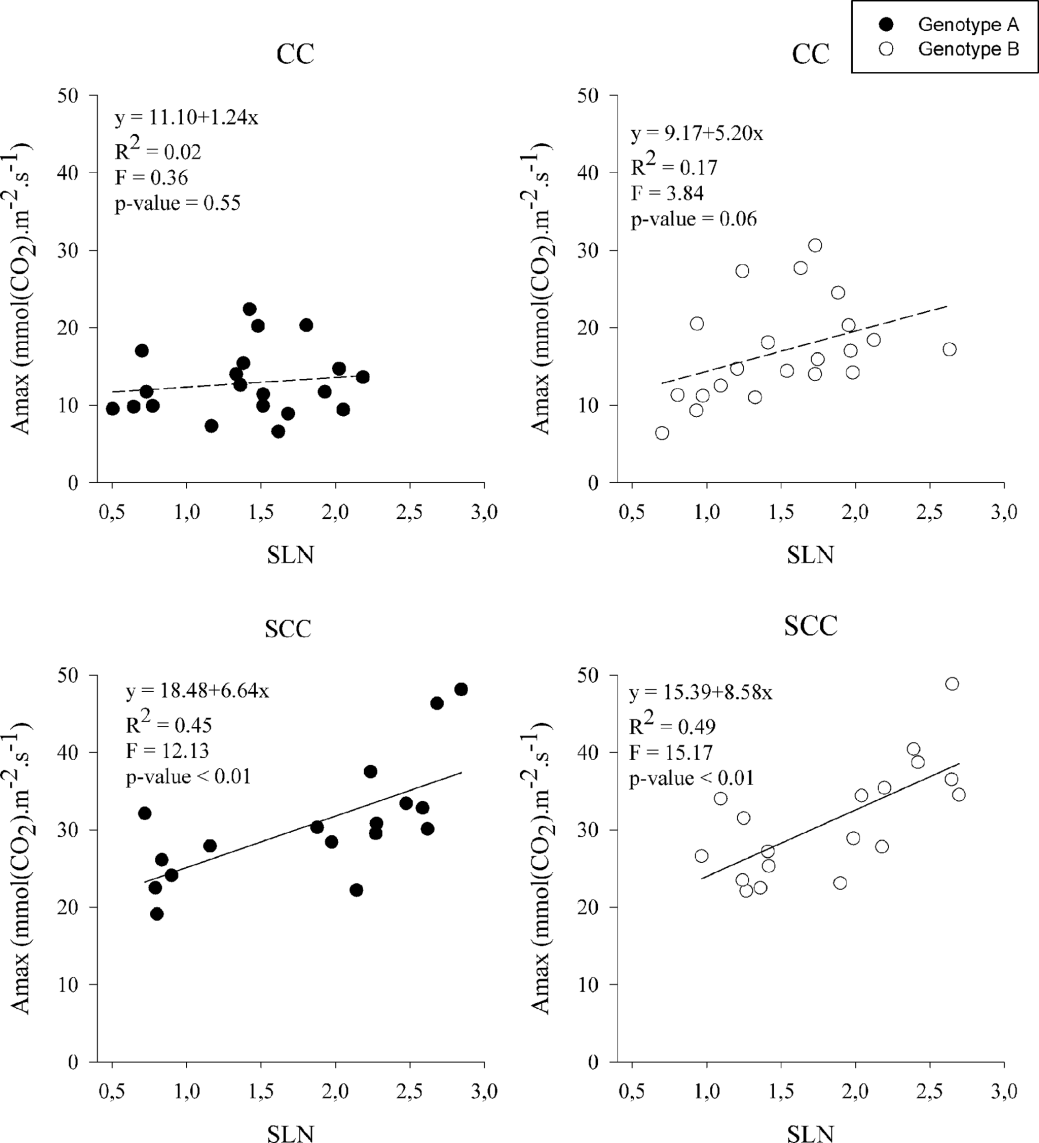
Relationship between specific leaf nitrogen (SLN, g.m^-2^) and light-saturated photosynthetic rates (Amax, μmol.m-².s^-1^) for genotypes A and B in controlled (CC) and semi-controlled conditions (SCC).

The maximum net rate of CO_2_ assimilation (*A*_*max*_) may be limited not only by light availability but also by leaf N content (per unit leaf area, i.e., SLN) [36]. No relationship was found between *A*_max_ and SLN under CC, where the mean PPFD was of 400 μmol m^−2^ s^−1^ (Fig 4). *A*_max_ was positively correlated to SLN under SCC, where the PPFD was comprised between 1000 and 2000 μmol m^−2^ s^−1^ (Fig 4).

### Steviol glycosides composition

**Fig 5:**
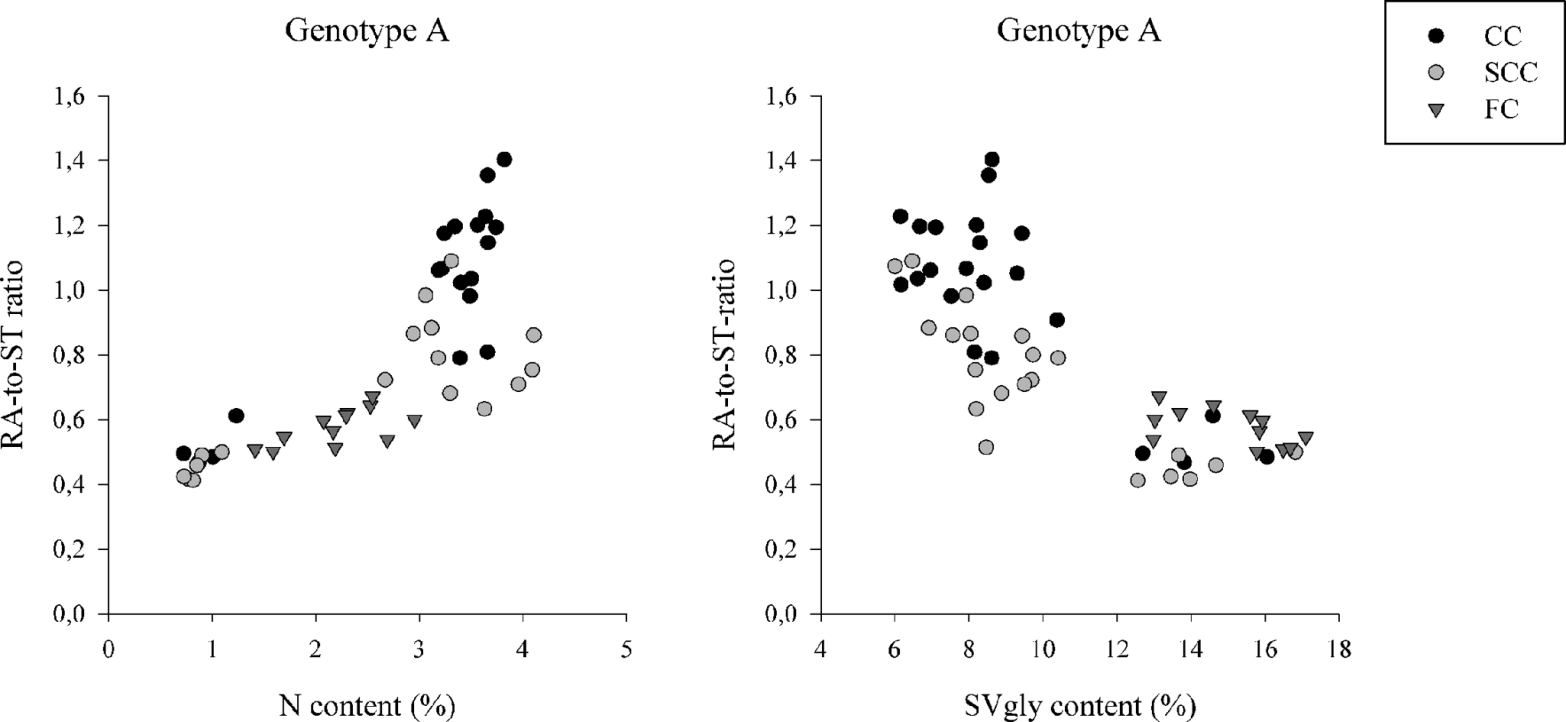
Relationship between nitrogen content in leaves (N content, %) and RA-to-ST ratio and relationship between steviol glycoside content in leaves (SVgly content, %) and RA-to-ST ratio for genotype A in controlled (CC), semi-controlled (SCC) and field conditions (FC).

Genotype B did not produce RA. For this genotype, N-supply level had no effect on ST proportion (% total content) under CC and SCC. Under FC, ST proportion was significantly lower on site III (Table 5). Regarding genotype A (RA producer), under CC and SCC, the proportion ST was significantly higher and that of RA significantly lower as SVgly content increased and N content decreased (Table 5 and Fig 5). Under FC, the proportion of ST was significantly higher as SVgly content increased and N content decreased but no significant differences were obtained for the proportion of RA (Table 5 and Fig 5).

**Table 5:** SVgly proportions (mean ± SEM with SEM calculated from the ANOVA residual mean square with 28 df under CC, 32 df under SCC and 16 df under FC) as affected by genotype (G) = A and B and nitrogen supply (N) = N1, N2, and N3 or location site (LS) = I, II, III, IV.

Growth conditions (GC)	Genotype (G)	Nitrogen supply (N) or Location site (LS)	ST proportion (%)	RA proportion (%)	Minor SVgly proportion (%)	*N*
CC	A	N1	52.8 ± 1.5	33.8 ± 1.9	13.4 ± 0.8	5
		N2	40.9 ± 1.3	44.7 ± 1.6	14.4 ± 0.7	7
		N3	41.0 ± 1.3	44.7 ± 1.6	14.3 ± 0.7	7
	B	N1	85.8 ± 1.4	0.0	14.2 ± 0.8	6
		N2	86.2 ± 1.2	0.0	13.8 ± 0.7	8
		N3	86.0 ± 1.3	0.0	14.0 ± 0.7	7
SCC	A	N1	58.7 ± 1.3	28.7 ± 1.6	12.6 ± 0.7	7
		N2	44.5 ± 1.2	40.0 ± 1.5	15.5 ± 0.7	8
		N3	51.1 ± 1.5	35.0 ± 1.9	13.9 ± 0.8	5
	B	N1	89.6 ± 1.2	0.0	10.4 ± 0.7	8
		N2	87.8 ± 1.2	0.0	12.2 ± 0.7	8
		N3	85.9 ± 1.2	0.0	14.1 ± 0.7	8
FC	A	I	56.5 ± 0.8	29.3 ± 0.8	14.3 ± 0.9	3
		II	55.6 ± 0.8	31.0 ± 0.8	13.4 ± 0.9	3
		III	49.9 ± 0.8	32.0 ± 0.8	18.0 ± 0.9	3
		IV	52.2 ± 0.8	30.6 ± 0.8	17.2 ± 0.9	3
	B	I	84.6 ± 0.8	0.0	15.4 ± 0.9	3
		II	83.2 ± 0.8	0.0	16.7 ± 0.9	3
		III	80.0 ± 0.8	0.0	20.1 ± 0.9	3
		IV	83.6 ± 0.8	0.0	16.2 ± 0.9	3

*N* represents the number of plants under CC and SCC, and the number of plots under FC. CC, controlled conditions; SCC, semicontrolled conditions; FC, Field conditions.

## Discussion

The present study focused on growth and SVgly accumulation in two contrasted *S*. *rebaudiana* genotypes under different growth conditions (CC, SCC, FC) and with different levels of N fertilization. Plants grown under CC were under suboptimal light intensity (PPFD 400 μmol m^−2^ s^−1^). The same experiment was replicated under non limiting natural light intensity in SCC (PPFD 1000–2000 μmol m^−2^ s^−1^). The experiment under field conditions (FC) was used to validate the CC and SCC observations under more realistic conditions of production. Whatever the growth conditions were, low N content in leaves was correlated to a decrease in leaf biomass and an increase in SVgly content (Fig 3). Moreover, this relationship was globally maintained in both genotypes.

Under CC and SCC, SLN was decreased by as much as 50%, whereas photosynthetic activity was less impaired. *A*_max_ decreased by 25% under CC and by 13%–32% under SCC. This suggests that, when N availability is limited, carbohydrate allocation promotes the accumulation of SVglys, which is consistent with the carbon-nutrient-balance hypothesis [24, 26]. The SVgly pathway is very active in the leaves of *S*. *rebaudiana*: in our study SVglys accumulated in leaves at concentrations ranging from 6% to 18% on a dry-mass basis and they may accumulate up to 30% [8]. Thus, a very large part of the *S*. *rebaudiana* metabolism is committed to the synthesis of these molecules. In contrast, gibberellins such as GA_20_ are present in *S*. *rebaudiana* leaves at concentrations of 1.2 μg/kg fresh biomass, which is 1/10,000 times less than SVglys [40]. SVgly pathway might be in competition with other pathways issued from the MEP, that lead to the synthesis of key isoprenoids, such as gibberellins, chlorophylls, and carotenoids. Considering the involvement of photosynthesis and NO_3_^−^ availability in regulating the MEP pathway [23], this pathway may be diverted from the synthesis of major components, such as chlorophylls, to the synthesis of SVglys when N is limited.

Plants responded to the low light intensity under CC (PPFD 400 μmol m^−2^ s^−1^) by decreasing *A*_max_, in comparison to those grown under SCC (PPFD 1000–2000 μmol m^−2^ s^−1^). This disrupted the relationship between *A*_max_ and SLN and thus, despite increasing N-supply, no differences were observed in plant biomass under CC. In consequence, this limiting condition was expected to lower the C/N ratio and to lead to a decrease in C-based secondary metabolites [28]. However, for both genotypes, no differences were obtained in SVgly content between CC and SCC (Table 3). Further investigations are necessary to establish the effect of light intensity on the MEP pathway and on SVgly accumulation in *S*. *rebaudiana* leaves.

The increase in SVgly content generally compensated the decrease in leaf biomass under controlled conditions (CC and SCC). However, under field conditions this effect was not sufficient, and the higher yield of SVglys (g pl^-1^) was achieved when environmental conditions favored biomass accumulation. The LMR is also a valuable parameter for improving leaf yield and thus *S*. *rebaudiana* productivity. Previous studies showed that there are useful levels of genotypic variability for this parameter [2]. In the present study, the LMR, was mainly affected by genotype, but this component tended to decrease as plant biomass increased.

SVgly composition is a determining factor of the quality of *S*. *rebaudiana* extracts, and this can be assessed by using the RA-to-ST ratio. For the RA-containing genotype (A), the RA-to-ST ratio decreased with increased SVgly accumulation (Fig 5). This result suggests that the activity of UGT76G1, which facilitates the conversion of ST to RA, is restricted to a constant but undetermined level (cf. Fig 1). Previous studies indicated the importance of enzymes that, to regulate SVgly synthesis, act prior to the formation of steviol [8, 17, 41–43]. However, the limiting activity of glycosyltransferases acting on the proportion of individual SVglys is less clear. Transition from long days to short days has been shown to accelerate the accumulation of RA and thus to increase the RA-to-ST ratio, in parallel with an up-regulation of UGT74G1 and UGT76G1 [43]. RA might also be formed from rebaudioside B through the activity of UGT74G1 [18]. In general, it is difficult to correlate a specific substrate with a specific derivative, because glycosyltransferases are thought to be highly region specific rather than substrate specific [44]. Thus, determining the best level of N supply to optimize SVgly content and composition depends on a better understanding of its effect on UGTs activity.

This study has shown that *S*. *rebaudiana* can respond adaptively to low N assimilation by decreasing its growth rate while increasing the SVgly content in its leaves. However, in field the main parameter for the SVgly yield remains the biomass production, favored by N supply. In more sustainable agricultural systems, this relationship could also be considered to find the best compromise between leaf biomass and SVgly concentration. In addition, the extraction of SVglys in more concentrated leaves implies less waste water. SVgly composition might also be affected, albeit in a lesser extent, by low N content in the leaves: the RA-to-ST ratio tended to decrease with increasing SVgly accumulation, suggesting a limiting role of this step in the glycosylation pathway.
